# Microstructured zirconia surfaces modulate osteogenic marker genes in human primary osteoblasts

**DOI:** 10.1007/s10856-014-5350-x

**Published:** 2015-01-13

**Authors:** Claudia Bergemann, Kathrin Duske, J. Barbara Nebe, André Schöne, Ulrike Bulnheim, Hermann Seitz, Jens Fischer

**Affiliations:** 1 Department of Cell Biology, University Medical Center Rostock, Schillingallee 69, 18057 Rostock, Germany; 2 VITA Zahnfabrik, H. Rauter GmbH & Co.KG, Spitalgasse 3, PO Box 1338, 79713 Bad Säckingen, Germany; 3 Institute for Dental Materials and Engineering, University of Basel, Hebelstrasse 3, 4056 Basel, Switzerland; 4 Fluid Technology and Microfluidics, University of Rostock, Justus-von-Liebig Weg 6, 18057 Rostock, Germany

## Abstract

In dentistry, zirconia has been used since the early 1990s for endodontic posts, more recently for implant abutments and frameworks for fixed dental prostheses. Zirconia is biocompatible and mechanically strong enough to serve as implant material for oral implants. Although several zirconia implant systems are available, currently the scientific and clinical data for zirconia implants are not sufficient to recommend them for routine clinical use. Here the influence of microstructured yttria-stabilized zirconia (YZ) on human primary osteoblast (HOB) behavior was determined. YZ surfaces were treated by sandblasting (YZ-S), acid etching (YZ-SE) and additionally heat treatment (YZ-SEH). Morphological changes of HOB were determined by scanning electron microscopy. Actin cytoskeleton was investigated by laser scanning microscopy and analyzed by novel actin quantification software. Differentiation of HOB was determined by real time RT-PCR. Improved mechanical interlocking of primary HOB into the porous microstructure of the acid etched and additionally heat treated YZ-surfaces correlates with drastically increased osteocalcin (OCN) gene expression. In particular, OCN was considerably elevated in primary HOB after 3 days on YZ-SE (13-fold) as well as YZ-SEH (12-fold) surfaces. Shorter actin filaments without any favored orientation on YZ-SE and YZ-SEH surfaces are associated with higher roughness (R_a_) values. Topographically modified yttria-stabilized zirconia is a likely material for dental implants with cell stimulating properties achieving or actually exceeding those of titanium.

## Introduction

The objective of oral implantology is to replace lost natural teeth with artificial, specifically designed implants with the purpose of providing additional masticatory units. The benefits of implants are (i) to avoid grinding of intact, adjacent teeth, which is unavoidable when constructing a fixed dental prosthesis, (ii) to avoid a removable partial denture by providing an abutment for a fixed restoration, or (iii) to stabilize a removable dental prosthesis. Titanium implants are state of the art. It is a generally and worldwide-accepted doctrine that the endosseous part consists of a screw to afford primary stability and a rough surface to guarantee the successful osseointegration. The part penetrating the mucosa has to have a polished surface to impede bacterial adhesion. These facts are undisputed and well established in the relevant textbooks [1]. Titanium implants have been used successfully for over 3 decades [2–14] and numerous publications attest to the success of implant-supported single crowns and fixed prostheses [10]. Complications may be an early failure, i.e. implant loss in the first weeks after insertion, or a late failure due to periimplantitis, i.e. loss of osseointegration after years of clinical service [1]. Surface modifications are created by sandblasting, plasma spraying or etching to accelerate osseointegration [15, 16].

To overcome the disadvantages of metallic implants, a ceramic implant may be considered a viable alternative. In dentistry, zirconia has been used since the early 1990s for endodontic posts [17], more recently for implant abutments [18, 19] and frameworks for fixed dental prostheses [20, 21]. Based on these results, it is obvious that zirconia may be applied as material for implants as well. It is undisputed in the literature that zirconia is biocompatible and mechanically strong enough to serve as implant material for oral implants [22–24]. The superior mechanical strength of zirconia, particularly in the event of tensile stress, originates from two effects. On the one hand the binding energy between Zr and O is high, which requires strong forces to break the bond. On the other hand zirconia is reinforced by adding yttria, which stabilizes the tetragonal high temperature phase. Tensile stress may trigger the suppressed phase transition from tetragonal to monoclinic even at room temperature. The change in crystal structure is associated with a volume increase of 3–5 %, establishing an intrinsic compressive stress, which counterbalances the tensile stress and thus protects the ceramic from its destructive effect. Although several yttria-stabilized tetragonal zirconia polycrystalline ceramic (Y-TZP) implant systems are available, currently the scientific clinical data for these zirconia implants are not sufficient to recommend them for routine clinical use [22]. Long-term stability, substantial osseointegration and a healthy transmucosal barrier are crucial prerequisites for dental implants. The osseointegration of an implant material is determined by the surface characteristics of the material like surface chemistry, surface charge, bulk material rigidity and roughness. These characteristics affect the adsorption of proteins from the extracellular matrix, thus governing cell adhesion to the material [25]. Optimal cell adhesion is in turn a prerequisite for the proliferation and differentiation of anchorage-dependent cells like bone cells, and for the stable integration of an implant into the surrounding tissue [26–29]. It is already known that surface roughness has a high impact on cell behavior at the cell-material interface [30–32].

In a review paper, Wenz et al. [24] reported on observation periods of Y-TZP osseointegration in animal models ranging from 4 weeks to 24 months. Bone-to-implant contact was above 60 % in almost all papers, which indicated successful osseointegration. In groups where titanium was used as a control, Y-TZP implants were at least similar to titanium. Moderately roughened (R_a_ ~ 1.5 µm) surfaces of Y-TZP showed fourfold to fivefold higher resistance to torque than machined surfaces. Independent of the implant material, the adherence of osteoblasts on rough surfaces is more pronounced than on smooth ones, which also applies to Y-TZP [33]. The results of bone-to-implant contact and push-in forces were similar for surfaces and independent of the material.

The main goal of this in vitro study was to determine the influence of microstructured yttria-stabilized zirconia surfaces treated by sandblasting, acid etching and heat treatment on human primary osteoblast behavior. Initial spreading, actin organization and cell differentiation on the mRNA level were evaluated.

## Materials and methods

### Sample characterization

As test material, disks of yttria-stabilized zirconia (YZ) with a diameter of 13 mm and a thickness of 2 mm were used. For micro-structuring, the surface of YZ specimens was sandblasted (YZ-S) (P-G 400, Harnisch & Rieth, Winterbach, Germany) with 105 µm alumina particles (Hasenfratz no. 120, Assling, Deutschland) at a pressure of 6 bar. The distance between nozzle and specimen surface was 18 mm. After sandblasting, specimens were etched with 40 % hydrofluoric acid, for 1 h (YZ-SE) and thoroughly rinsed in distilled water. The following heat treatment of sandblasted, acid-etched specimens (YZ-SEH) was performed for 3 h at 1,250 °C (LH 15/14, Nabertherm, Lilienthal, Germany). The arithmetical mean roughness (R_a_) of the specimens after sandblasting, acid etching and heat treatment was measured with the Hommel Wave System (Hommel Wave, VS-Schwenningen, Germany). To determine differences in the nanostructure of the treated YZ-surfaces confocal laser scanning microscopy (LEXT OLS4000, Olympus, Tokyo, Japan) was used. Line roughness values were acquired with a magnification of ×50 and a threshold wavelength λ_c_ of 25 µm. λ_c_ is the factor that eliminates waviness from roughness. The threshold wavelength thus represents a filtering of the primary profile. The scanning area of the specimens was 259 × 259 µm. Five line scans per area and three areas per surface were evaluated (n = 15). YZ-specimens without sandblasting, acid etching and heat treatment as well as titanium of technical purity (grade 2, 11 mm in diameter) modified by machining (Ti-M) were taken for comparison. Before their use in experiments, all materials were sterilized with 70 % ethanol and dried in a safety cabinet.

### Cell biological investigations

#### Cell culture

Human primary osteoblasts (HOB, C-12720, LotNr. 9010802.1, Promocell, Heidelberg, Germany) were cultured in osteoblast growth medium with supplement mix (Promocell) and used in passages 4–7. Material samples were seeded with 1.5 × 10^4^ primary HOB cells and incubated at 37 °C in a humidified atmosphere with 5 % CO_2_ for appropriate time intervals.

#### Cell morphology with scanning electron microscopy (SEM)

After 24 h, cells were rinsed with phosphate buffered saline (PBS), fixed with 2.5 % glutaraldehyde (Merck, Darmstadt, Germany), dehydrated through a graded series of alcohol, dried in a critical point dryer (K850, EMITECH, Ashford, UK) and sputtered with gold (SCD 004, BAL-TEC, Leica Microsysteme, Wetzlar, Germany). The samples were characterized by scanning electron microscopy (SEM DSM 960A, Carl Zeiss, Jena, Germany).

#### Cell spreading

To make the cells visible for microscopic investigations on the opaque material, HOB were labeled with the red fluorescent dye PKH26 for vital cells (PKH26-GL general cell linker kit, Sigma Aldrich, Munich, Germany) before seeding. This fluorescent dye did not influence the cell growth of osteoblasts, and the sensitivity of the PKH26-stained cells to topographical and chemical features of material surfaces was maintained, as seen in earlier experiments [31, 34]. The cell membranes of 1 × 10^5^ suspended cells (in 250 µl diluent C according to the kit instructions) were stained with PKH26 for 5 min at 37 °C using a dilution of 2 µl PKH26 + 248 µl diluent C. 1.5 × 10^4^ labeled HOB cells were seeded onto the samples. After 24 h, cells were rinsed in PBS, fixed with 4 % paraformaldehyde (PFA) (Merck) for 10 min at room temperature (RT) and were embedded with mounting medium [34] and a cover slip. Cells were examined by confocal laser scanning microscopy (LSM 410, exc. 543 nm, Carl Zeiss) and the spreading (cell area in µm^2^) of 40 cells/specimen was then analyzed using the software ‘area measurement’ of the confocal microscope. Two separate experiments with different cell passages were done for each sample (n = 80 cells).

#### Actin cytoskeleton

The actin cytoskeleton of HOB cells was stained as already described [31, 35]. Briefly, after 24 h cells were rinsed with PBS, fixed with 4 % PFA for 10 min at RT, washed with PBS again, permeabilized with 0.1 % Triton X-100 (Merck, 10 min, RT) and incubated with BODIPY FL phallacidin (diluted 1:40, Invitrogen) at RT in the dark for 30 min. Cells were embedded and examined by LSM with a Plan-Apochromat 63×/1.40 Oil DIC M27 objective. Overlay images of z-scans were assembled by scanning the cells on the surface at 0.8 µm intervals (software ZEN, Carl Zeiss). The actin cytoskeleton was quantified by means of our recently developed, novel software FilaQuant [36, 37]. At least eight images of the actin cytoskeleton of HOB cells on each surface were analyzed for average filament length, total filament length, maximum filament length and filament orientation dispersion.

#### Gene expression

HOB cells were seeded onto the samples (two disks each) and cultured for 1 or 3 days. Total RNA was isolated using the NucleoSpin RNA kit (Macherey-Nagel, Düren, Germany). First-strand cDNA was synthesized from at least 200 ng total RNA by reverse transcription with SuperScript II (Invitrogen) using 2.5 µM random hexamers (Invitrogen). Quantitative real-time PCR assays were performed and monitored using the ABI PRISM® 7500 sequence detection system (Applied Biosystems, Darmstadt, Germany). The PCR reactions contained 4 µl diluted cDNA (2.5-fold) in a reaction volume of 20 µl, 1× TaqMan® Universal PCR Master Mix (Applied Biosystems) and 1 µl assays-on-demand gene expression assay mix (Applied Biosystems) for the detection of alkaline phosphatase (ALP, #Hs00758162_m1ALPL), collagen type 1 (COL I, #Hs00164004_m1COLA1), osteocalcin (OCN, #Hs01587813_g1BGLAP) and for glyceraldehyde 3-phosphate dehydrogenase as an endogenous control (GAPDH, #Hs99999905_m1GAPDH, housekeeping gene). In general, for gene expression analysis PCR amplification was performed in triplicate and repeated in three independent experiments. Gene expression values were calculated based on the comparative ΔΔCT-method, normalized to GAPDH as an endogenous control (housekeeping gene) and calibrated to Ti-M at 1 day.

### Statistics

Statistical analysis of data sets was performed using the software SPSS 15.0 for Windows (SPSS Inc., Chicago, IL, USA). Data are expressed as mean values ± standard deviation and analyzed using the Mann-Whitney U test or ANOVA and posthoc Bonferroni test. The results of the gene expression studies were analyzed using the Mann-Whitney U test and values were compared to Ti-M at the same time point. Differences for all experiments were considered statistically significant at *P* < 0.05 (*P < 0.05, ^#^ *P* < 0.01, ^+^ *P* < 0.001).

## Results

### Characterization of materials

Table 1 demonstrates the surface roughness (R_a_, arithmetical mean deviation of the profile) of the ceramic specimens determined by the Hommel Wave System. The machined surface showed a roughness of 0.59 µm, which increased by the factor of 2 after sandblasting. Acid etching slightly raised the R_a_ value, but the final heat treatment did not significantly change the roughness values measured with the Hommel Wave System any more. To identify differences in the nanostructure of the treated surfaces, we used confocal laser microscopy with a threshold wavelength of 25 µm to get values for the roughness eliminated from waviness. By this method we could show that the heat treatment does finally reduce the surface roughness in the nanoscale (see Table 2).

**Table 1:** Arithmetic mean roughness values determined by the Hommel wave system

	R_a_ (µm)
Zirconia machined (YZ)	0.59
Zirconia sandblasted (YZ-S)	1.22
Zirconia sandblasted and acid etched (YZ-SE)	1.31
Zirconia sandblasted, acid etched and heat treated (YZ-SEH)	1.32
Titanium machined (Ti-M)	0.54

**Table 2:** Roughness values determined by confocal laser microscopy

Roughness (μm)	YZ	YZ-S	YZ-SE	YZ-SEH
R_a_	0.07	0.32	0.35	0.36
R_t_	1.15	3.08	4.48	4.16
R_z_	0.46	1.55	2.10	2.05
R_q_	0.09	0.39	0.45	0.45

Data reflect roughness eliminated from waviness due to a λ_c_-filter of 25 µm

*R*_*a*_ arithmetic mean roughness values, *R*_*t*_ maximum peak-to-valley height, *R*_*z*_ arithmetic average of the maximum peak-to-valley height of the five greatest values, *R*_*q*_ standard deviation of height amplitude

**Fig. 1:**
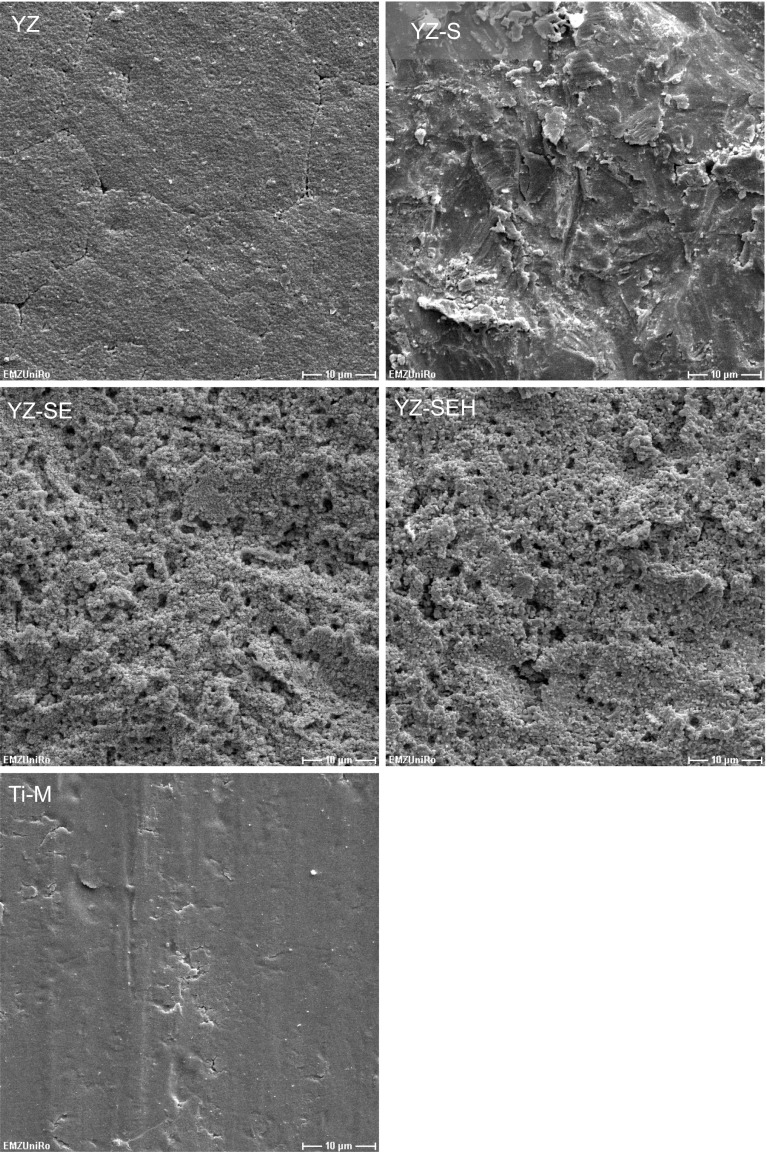
Surface topography of yttria-stabilized zirconia after sandblasting (YZ-S), acid etching (YZ-SE) and heat treatment (YZ-SEH) in comparison to machined titanium (Ti-M). (SEM DSM 960A, Carl Zeiss, magnification ×2,000, *scale bars* 10 µm)

Scanning electron microscopy attested the increasing surface roughness of YZ according to the treatments used (Fig. 1). YZ and Ti-M are relatively smooth surfaces with striations. Sand blasting (YZ-S) results in sharper edges and indentations, additional acid etching (YZ-SE) and tempering (YZ-SEH) produce a more porous structure. The pores of different sizes are regularly distributed over the whole surface.

### Cell morphology and spreading

**Fig. 2:**
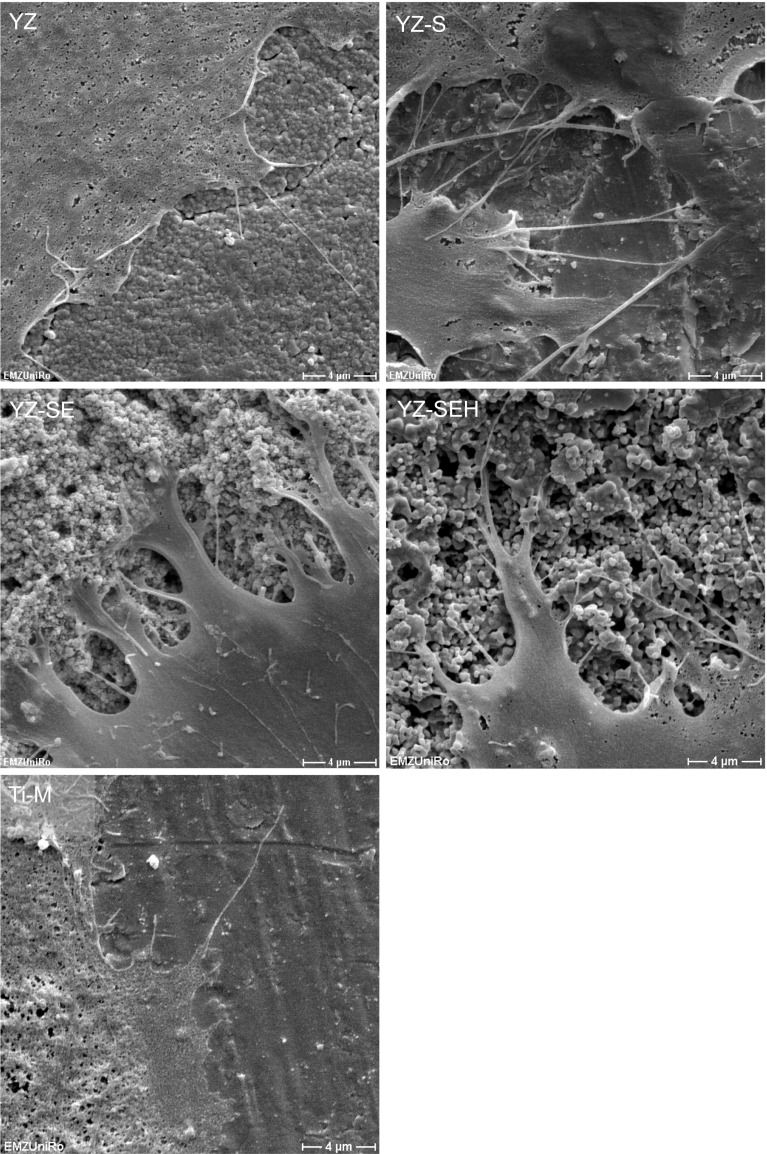
Primary HOB cell morphology on zirconia samples after 24 h. Note that cells on all surfaces demonstrate a well-spread morphology and model the topography of the rough porous surfaces on YZ-SE and YZ-SEH. (SEM DSM 960A, Carl Zeiss, magnification ×5,000, *scale bars* 4 µm)

**Fig. 3:**
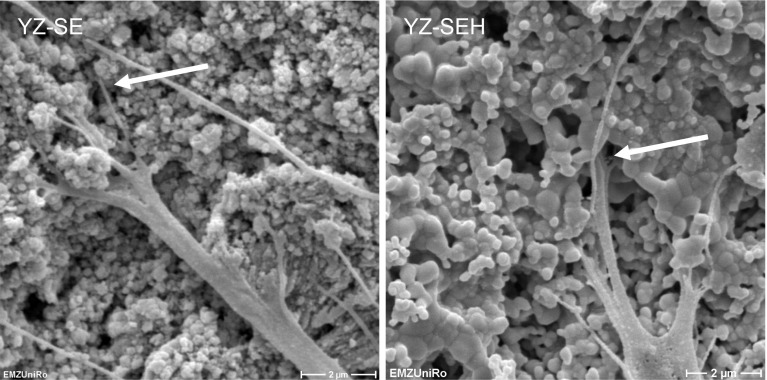
Magnified view on cell’s filopodia interlocking (*arrows*) into the porous surfaces of acid etched YZ-SE (*left*) and heat treated YZ-SEH (*right*) zirconia after 24 h. (SEM DSM 960A, Carl Zeiss, magnification ×10,000, *scale bars* 2 µm)

The SEM analysis of HOB (Fig. 2) after 24 h showed typically flat polygonal cells, which are regularly distributed on all YZ surfaces and Ti. Cells on YZ-S, YZ-SE and YZ-SEH seem to spread less compared to the controls YZ and Ti-M. In SEM images with a magnification higher than ×5000 it is visible that cells are more deeply anchored inside the rougher surfaces and mechanically interlocked (Fig. 3). Furthermore alterations in nanostructure could be detected and microparticles on the surface of YZ-SEH seem to be melted together.

**Fig. 4:**
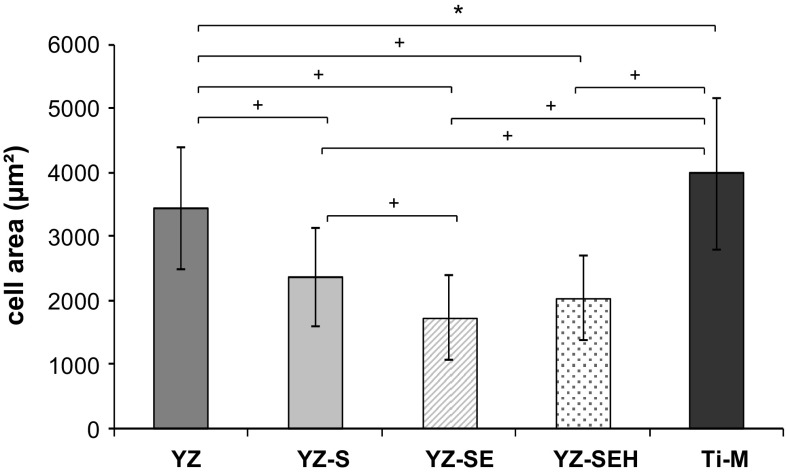
Spreading of primary HOB after 24 h. Note that cells expand to a less extent on the topographically modified surfaces YZ-S, YZ-SE and YZ-SEH compared to the untreated YZ as well as Ti-M. (LSM 410, Carl Zeiss, n = 80 cells, mean ± SD, *P < 0.05, ^+^ *P* < 0.001; ANOVA, posthoc Bonferroni)

In general, the cell areas on the microstructured samples with a higher roughness (YZ-S, YZ-SE, YZ-SEH) are reduced compared to controls. Although cells demonstrate a spread phenotype and are anchored with their filopods within the porous structures of YZ-SE and YZ-SEH, cell areas of HOB on these samples are significantly the smallest (Fig. 4). Heat treatment (YZ-SEH) after acid etching caused only a small effect on cell spreading.

### Actin cytoskeleton

**Fig. 5:**
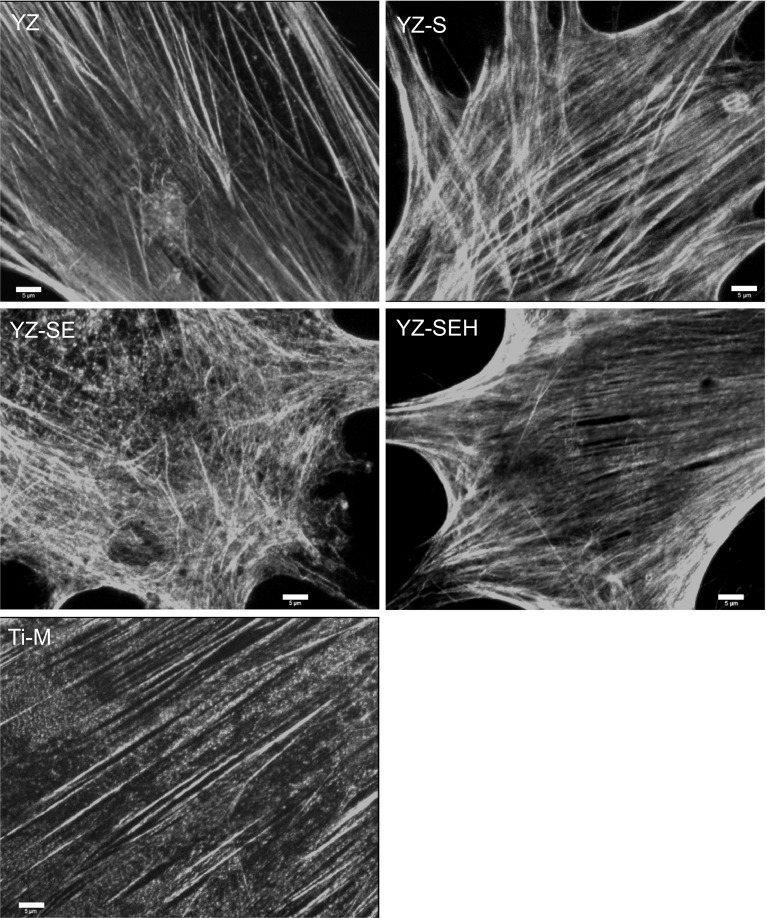
Actin cytoskeleton of primary HOB after 24 h. Note the ability of cells to sense the surface microtopography resulting in a rearrangement of actin fibers on the more rough porous YZ-SE and YZ-SEH. (LSM 410, exc. 543 nm, Carl Zeiss, *scale bars* 5 µm)

The cell’s actin cytoskeleton is important for cell migration, cell signaling and function. The organization of actin filaments in primary HOB was therefore investigated after 24 h on the rougher surfaces (YZ-S, YZ-SE and YZ-SEH) in comparison with controls (Ti-M, YZ) (Fig. 5). The actin filament length was quantified by means of our recently developed, novel software FilaQuant [36, 37]. Using this software we are able to quantify alterations of the actin network depending on the microtexture of the material surface. We observed typical long and straight actin stress fibers on Ti-M and untreated YZ. However, on the rough, microstructured modifications, the actin cytoskeleton appears to be organized in shorter stress filaments (Table 3).

**Table 3:** Actin filament quantification of primary HOB after 24 h

	
	YZ	YZ-S	YZ-SE	YZ-SEH	TiM
Total filament length (μm)	5480.2 ± 2184.5	5218.1 ± 1806.3	2949.0 ± 1713.6	4710.8 ± 2200.1	6676.9 ± 4161.2
Average filament length (μm)	36.6 ± 9.8	32.9 ± 5.2	22.1 ± 6.8^b,e,g,h^	29.4 ± 3.8^f^	37.6 ± 8.2
Maximum filament lengths (μm)	230.0 ± 71.2	197.9 ± 49.3	152.4 ± 75.5^f^	188.0 ± 64.6	258.7 ± 55.1
Orientation dispersion (°)	14.8 ± 3.8	19.3 ± 3.9^c,d^	21.6 ± 1.8^a,d^	17.7 ± 4.9^f^	12.0 ± 3.3

Note that actin filament length on YZ-S, YZ-SE and YZ-SEH surfaces is shortened due to the microstructure. Filament length values in cells on YZ-SEH are higher than on YZ-SE (Fluorescence microscopic images, scale bars 20 µm)

^a^ *P* < 0.001 versus YZ; ^b^ *P* < 0.01 versus YZ; ^c^ *P* < 0.05 versus YZ; ^d^ *P* < 0.001 versus Ti-M; ^e^ *P* < 0.01 versus Ti-M; ^f^ *P* < 0.05 versus Ti-M; ^g^ *P* < 0.01 versus YZ-S; ^h^ *P* < 0.01 versus YZ-SEH (mean ± SD, Mann-Whitney* U* test, n ≥ 8)

Actin fibers on YZ-S, YZ-SE and YZ-SEH are not only significantly shorter compared to YZ but also distributed irregularly which is reflected in the higher orientation dispersion values (0° would imply one preferred orientation and 28.65° indicates an undirected distribution). Interestingly, acid etching of YZ surfaces after sand blasting induced the shortest actin filaments in HOB cells whereas further heat treatment significantly elongated the cells’ actin filaments (average filament length 22.1 µm on YZ-SE and 29.4 µm on YZ-SEH). These data underline the visual impression taken from Fig. 5 that primary HOB cells show shortened actin filaments without any favored orientation on treated YZ surfaces, whereas heat treatment after sand blasting and acid etching seems to reverse this effect.

### Cell differentiation

**Fig. 6:**
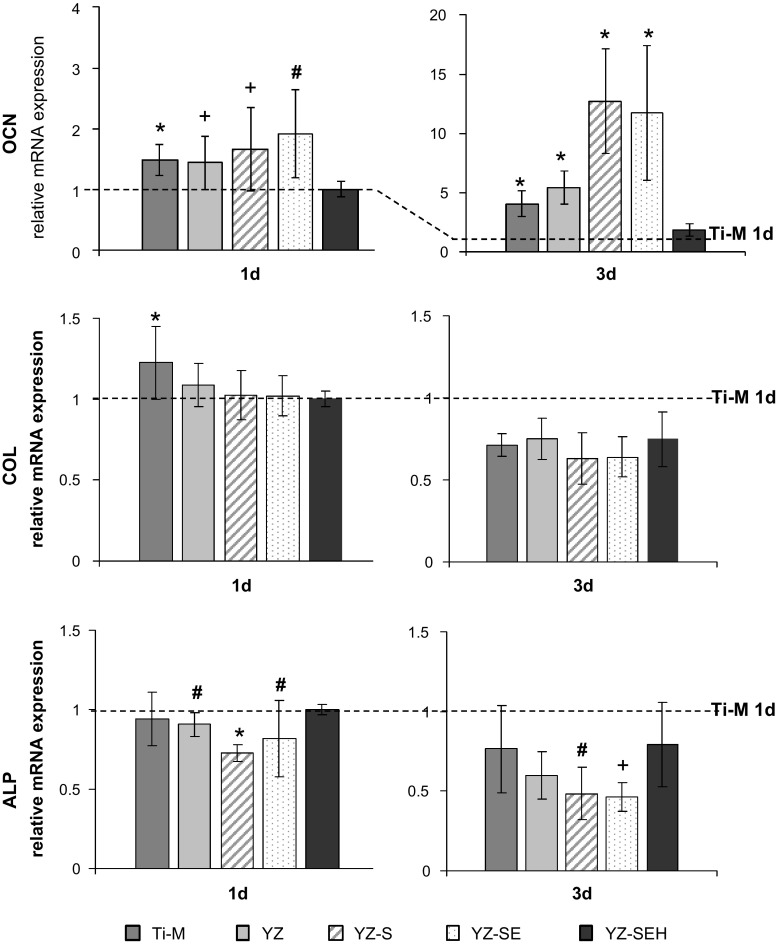
Relative gene expression of osteogenic differentiation proteins OCN, COL and ALP in primary HOB cells. Note that OCN gene expression is significantly higher on all ceramic surfaces, especially on the porous YZ-SE and YZ-SEH which is impressive at day 3 (n = 9, mean ± SD, *P < 0.05, ^#^ *P* < 0.01, ^+^ *P* < 0.001 related to Ti-M 1 day; Mann-Whitney* U* test)

The expression of osteogenic marker genes was investigated after 1 and 3 days for primary HOB cells: alkaline phosphatase (ALP), collagen I (COL), and osteocalcin (OCN) (Fig. 6). The gene expression values were normalized to glyceraldehyde 3-phosphate dehydrogenase (GAPDH) as a housekeeping gene and calibrated to Ti-M at 1 day. In general, expression of mRNAs encoding ALP as an early osteogenic marker was found to be down-regulated during the culture time, whereas the expression of OCN (marker for late stages of differentiation) increased. Significantly reduced ALP mRNA expression could be detected for primary HOB cells on YZ-SE and YZ-SEH surfaces already after 1 day and also after 3 days of culture. COL gene expression in primary HOB cells declined during culture and was after 1 day at the same level for YZ-SE and YZ-SEH as for Ti-M. Notably, OCN expression in primary HOB on all YZ samples was higher than on Ti-M. The mRNA level of OCN was considerably affected by the different surface treatments and was significantly increased in HOB cells on YZ-SE (13-fold) and YZ-SEH (12-fold) compared to untreated YZ after 3 days of culture (Fig. 6).

## Discussion

The clinical success of implant materials depends on their physical and chemical surface properties, which regulate the initial cell response. Optimal cell adhesion is in turn a prerequisite for proliferation and differentiation and contributes to osseointegration [26–28, 32]. Surface roughness has a high impact on the cell behavior at the material interface as shown for titanium [31, 38]. A few studies have reported on the effect of surface roughness (sand blasting) on ceramic materials like Y-TZP [30, 33, 39]. Sandblasting of Y-TZP creates sharp edged surfaces. By etching the sandblasted surface, the edges are rounded and pits are created. Such a surface topography is much closer to the surfaces successfully used with osseointegrating titanium implants. In this study, we focused on the response of primary human osteoblasts on yttria-stabilized zirconia surfaces with a microstructure created by etching after sand blasting. The surface roughness value R_a_ of our zirconia samples more than doubled after sandblasting (YZ-S), compared to the machined surface (YZ). Acid etching slightly enhanced not only the surface roughness (R_a_) but changed also topographical characteristic by rounding the sharp edges from sandblasting and creating small cavities. Heat treatment of sandblasted, acid-etched specimens generated a smoother nanotopography which is not reflected in the R_a_ values determined by the Hommel Wave System but in roughness values received by confocal laser scanning microscopy and in SEM images with high magnifications. Surfaces with an R_a_ ranging from 1 to 2 µm are reported to be optimal for cell attachment and osseointegration [40]. In the present study the roughness of the machined surface YZ as control was definitely below that range, while the roughness of all three microstructured surfaces ranged within the scope.

Our SEM images after 24 h demonstrate improved mechanical interlocking of primary HOB cells on rough, modified YZ samples especially for YZ-SE and YZ-SEH. On these surfaces osteoblasts developed long, very thin filopodia which seem to sense the surface for gaps where they can anchor. Our observations are supported by other in vitro studies reporting a significant increase in osteoblast adhesion on Y-TZP with increasing surface roughness for human CAL72 osteoblast cells [30] and mouse osteoblast-like cells [38]. In earlier cell-material interface studies we recognized the influence of surface roughness on adhesion components in primary human osteoblasts [31]. Cellular adhesion contacts were shortened on rough glass-blasted (R_a_ 1.22 µm) and corundum-blasted titanium surfaces (R_a_ 6.07 µm), actin filaments were disorganized and cell spreading was reduced. In agreement with these results derived from titanium, we found rearranged actin organization also on the three treated zirconia surfaces here, as well as a significantly reduced spreading of primary HOBs compared to untreated YZ surfaces. By means of the automatic actin quantification software FilaQuant we were able to quantify these microscopical observations. This showed that shorter actin filaments without any favored orientation correlate with higher R_a_ and R_t_ values generated by confocal laser microscopy. It is especially noteworthy that actin filaments were elongated on YZ-SEH compared to YZ-SE surfaces. In this respect it seems that heat treatment has an impact on the cytoskeleton organization of cells on these sandblasted etched zirconia and could be a special effect of the smoother nanosurface. Our findings appear to be in line with an earlier report, describing changes in the cytoskeleton of primary human jaw osteoblasts on sandblasted and acid etched titanium samples [41].

For the achievement of successful osseointegration of an implant osteoblasts should display adequate gene expression. Therefore it is important to understand gene regulation in the osteoblast linage. Billard et al. [42] investigated osteoblast cell maturation and studied gene expression of different osteogenic marker proteins for separate differentiation stages on tissue culture polystyrene. According to this human osteoblast differentiation model, COL and ALP are early differentiation markers in the osteoblast lineage and mRNA expression of these markers is high in preosteoblasts and declines during osteoblast maturation. On the other hand, OCN is a late differentiation marker and mRNA is expressed at very low levels first, but transcription is enhanced in later differentiation stages. It was shown on rough titanium surfaces in vitro that osteoblastic cells exhibited reduced cell proliferation and spreading but increased gene expression for osteogenic marker proteins as a sign of enhanced osteoblast maturation [38, 43, 44], indicating a positive microtopographical regulation on the mRNA level. Masaki et al. [45] were able to show enhanced ALP gene expression for human palatal mesenchymal cells cultured for 72 h on rough titanium surfaces (SLA-2, R_a_ 2.93 µm) and slight effects on other osteogenic differentiation markers like OCN and COL. In line with the osteoblast differentiation model of Billiard et al. [42] in our investigation, we found declined ALP and COL gene expression and increased gene expression for OCN in primary HOB cells in a time frame of 3 days as a sign of progressive osteoblast maturation. Furthermore, the primary HOB cells on modified YZ surfaces showed signs of an enhanced cell maturation compared to the untreated YZ and to the Ti control, i.e. (i) significantly down-regulated ALP mRNA expression on YZ-SE and YZ-SEH after 3 days, (ii) decreased COL mRNA on YZ-SE and YZ-SEH after 3 days, (iii) highly significant enhanced OCN gene expression on YZ-SE and YZ-SEH after 1 and 3 days of culture. So, significantly reduced cell areas on rough YZ-samples due to sandblasting, etching and heat treatment correlate with increased cell maturation in HOB cultured on these surfaces. Our results correspond with the studies of Martin et al. and Nebe et al. for reduced cell area but enhanced osteogenic gene expression on rough titanium [38, 43]. Elevated cell maturation was also found by Carinci et al. [46] in osteoblast-like MG-63 cells cultivated for 24 h on zirconia (ZrO_2_) samples and by Kohal et al. [47] within cells of the osteoblast cell line hFOB 1.19 after 21 days on processed Y-TZP surfaces too.

As a result of our study, for primary HOB cells it can be assumed that actin cytoskeleton is reorganized (shorter filaments) by increased surface roughness, but nevertheless cell differentiation seems to be stimulated. Higuchi et al. [48] also found a positive correlation between transient dynamic actin cytoskeletal changes and osteoblast differentiation. Short-term cytochalasin D treatment, resulting in fragmentation of the actin fibers, increased alkaline phosphatase activity, osteocalcin secretion and mineralization in MC3T3-E1 cells. Osteoblast differentiation has diverse trigger points and is influenced in a different way depending on the cell type, the culture time interval and the determination target. Therefore, more investigations regarding this topic are needed. For our human primary osteoblasts we observed a strong enhancement of osteocalcin mRNA level which was only topographically induced. Also Altmann et al. [49] found out that bioactivation of titanium- and zirconia-based materials via UV-functionalization appears to be not the main causative for the modulation of distinct cell functions in primary human alveolar bone osteoblasts, but were more governed by surface topography.

## Conclusion

Within the limitations of this study it can be concluded that yttria-stabilized zirconia is a likely material for dental implants with cell stimulating properties achieving or actually exceeding those of titanium. Yttria-stabilized zirconia, especially a sandblasted, acid etched and additionally heat-treated zirconia surface, is attractive for human primary osteoblasts. Treated surfaces induced reduced cell spreading, due to shortened actin filaments, combined with significant enhanced cell maturation and supported the mechanical interlocking of the cells into the porous microstructured interface.
